# Crocin-Phospholipid Complex: Molecular Docking, Molecular Dynamics Simulation, Preparation, Characterization, and Antioxidant Activity

**DOI:** 10.5812/ijpr-144041

**Published:** 2024-03-24

**Authors:** Yasaman Rezaee, Elham Rezaee, Leila Karami, Maryam Torshabi, Azadeh Haeri

**Affiliations:** 1 Department of Pharmaceutics and Pharmaceutical Nanotechnology, School of Pharmacy, Shahid Beheshti University of Medical Sciences, Tehran, Iran; 2 Department of Pharmaceutical Chemistry, School of Pharmacy, Shahid Beheshti University of Medical Sciences, Tehran, Iran; 3 Department of Cell and Molecular Biology, Faculty of Biological Sciences, Kharazmi University, Tehran, Iran; 4 Department of Dental Biomaterials, School of Dentistry, Shahid Beheshti University of Medical Sciences, Tehran, Iran; 5 Protein Technology Research Center, Shahid Beheshti University of Medical Sciences, Tehran, Iran

**Keywords:** Crocin, Phospholipid Complex, Molecular Docking, Molecular Dynamics Simulation, Solubility

## Abstract

**Background:**

Crocin is a water-soluble carotenoid compound present in saffron (*Crocus sativus* L.), known for its wide range of pharmacological activities, including cardioprotective, hepatoprotective, anti-tumorigenic, anti-atherosclerosis, and anti-inflammatory effects.

**Objectives:**

The instability of crocin, its low miscibility with oils, and poor bioavailability pose challenges for its pharmaceutical applications. This study aimed to design and prepare a crocin-phospholipid complex (CPC) and assess its physicochemical properties.

**Methods:**

The study investigated the formation of the complex and its binding affinity through molecular docking. Molecular dynamics (MD) simulations were conducted to find the optimal molar ratio of crocin to phospholipid for the complex's preparation. The CPC was produced using the solvent evaporation method. Techniques such as X-ray diffraction (XRD), Fourier-transform infrared spectroscopy (FTIR), field-emission scanning electron microscopy (FE-SEM), nuclear magnetic resonance (NMR), and solubility studies were utilized to characterize and confirm the formation of CPC. Additionally, the in vitro antioxidant activity of crocin and CPC was evaluated.

**Results:**

Molecular dynamic simulations explored molar ratios of 1: 1, 1: 1.5, and 1: 2 for crocin to phospholipid. The ratio of 1: 2 was found to be the most stable, exhibiting the highest probability of hydrogen bond formation. Molecular docking, FTIR, and NMR studies indicated hydrogen bond interactions between crocin and phospholipid, confirming CPC's formation. XRD and FE-SEM analyses showed a decrease in crocin's crystallinity within the phospholipid complex. Furthermore, the solubility of crocin in n-octanol was enhanced post-complexation, indicating an increase in crocin's lipophilic nature.

**Conclusions:**

Phospholipid complexation emerges as a promising technique for enhancing the physicochemical characteristics of crocin.

## 1. Background

**Figure 1.**
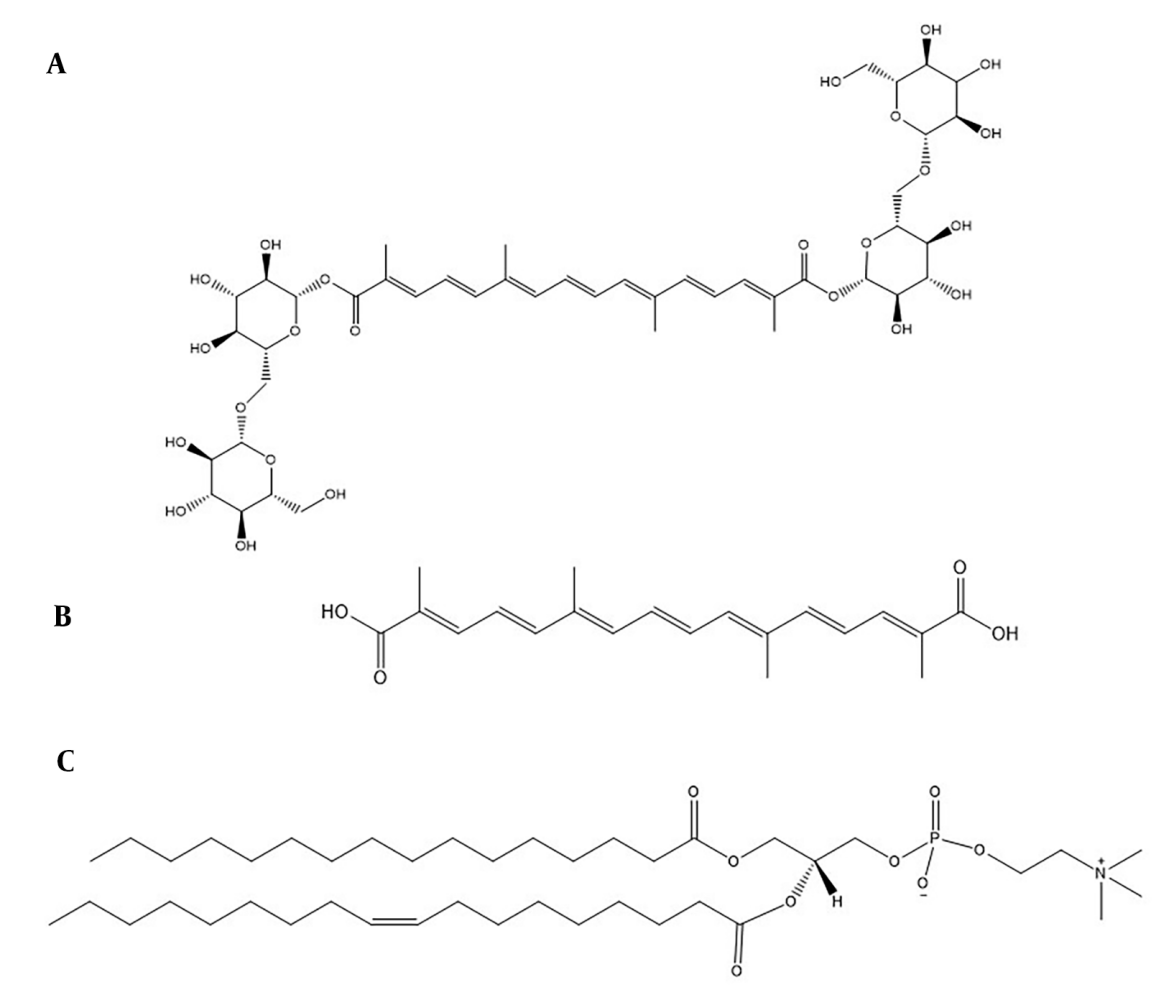
Chemical structure of A, crocin; B, crocetin, and ;C, phospholipid (EPC).

Crocin, a natural carotenoid extracted from saffron, is found in the dried stigmas of *Crocus sativus* L. flowers. Chemically, it is a glycosylated ester of crocetin, characterized by high water solubility due to its sugar moieties (Figure 1A) (1). Recent studies have highlighted crocin's diverse medicinal properties, including cardioprotective, hepatoprotective, anti-tumorigenic, anti-atherosclerotic, neuroprotective, and anti-inflammatory effects (2, 3). However, crocin's intravenous administration results in lower plasma levels of crocetin (Figure 1B), and its oral bioavailability is compromised by low penetration and hydrolysis to crocetin, suggesting transformation in the gastrointestinal tract (4, 5). Therefore, there is a need to develop an effective delivery system to enhance crocin's physicochemical properties and overall efficacy. Previous research has explored various strategies, such as polymeric particles (6), nanoemulsions (7), liposomes (8), and niosomes (9).

The application of lipids in drug delivery systems has gained prominence recently, offering improved physicochemical properties, enhanced oral bioavailability, and reduced toxicity when therapeutic agents are conjugated with lipids. Fatty acids, steroids, glycerides, and phospholipids have been employed to create lipid-drug conjugates (10). Phospholipids, in particular, play a crucial role in drug delivery. As a key component of cell membranes, this amphiphilic lipid can form lipid bilayers. The amphiphilic nature of phospholipids, with a phosphate head and two hydrophobic tails (Figure 1C), allows for the formation of phospholipid-based complexes through covalent or non-covalent interactions, potentially leading to enhanced oral bioavailability and reduced drug leakage (11-13). The technology of drug-phospholipid complexes has been successfully applied in recent years to improve the characteristics of natural substances, including chrysin (14), piperine (15), and luteolin (16). However, to the best of our knowledge, this study represents one of the first attempts to utilize computer-aided design and molecular dynamics (MD) simulation studies for the optimized preparation of phospholipid-drug conjugates. Additionally, there have been no previous reports on the preparation and characterization of a crocin-phospholipid complex (CPC).

## 2. Objectives

The aim of this study was to develop a phospholipid complex of crocin for the first time. A molecular docking study was conducted to examine the affinity and nature of physicochemical interactions between crocin and the phospholipid. The MD simulation method was utilized to explore the interactions between crocin and the phospholipid across various molar ratios, with the goal of identifying the optimal crocin-to-phospholipid molar ratio. The CPC was synthesized using the solvent evaporation technique, and its apparent solubility was measured. The physicochemical characteristics of the CPC were analyzed through X-ray diffraction (XRD), Fourier-transform infrared spectroscopy (FTIR), field emission scanning electron microscopy (FE-SEM), and nuclear magnetic resonance (NMR) spectroscopy. Additionally, the antioxidant properties of both crocin and the CPC were evaluated to determine any changes resulting from the complexation process.

## 3. Methods

### 3.1. Materials

Crocin, dichloromethane, vitamin C, and diphenylpicrylhydrazyl (DPPH) were acquired from Merck/Sigma–Aldrich (Germany). Egg phosphatidylcholine (EPC) was sourced from Lipoid GmbH (Ludwigshafen, Germany). All other reagents and chemicals used were of analytical grade.

### 3.2. In Silico Investigation of Complexation

#### 3.2.1. Molecular Docking

The chemical structures of crocin and the phospholipid (EPC) were retrieved from PubChem and illustrated using ChemDraw^®^ 18.2. The 2D structures were then converted to 3D, and their energy was minimized using the MM2 force field within Chem3D Ultra^®^ 18.2. Autodock^®^ Tools version 1.5.6rc3 (http://mgltools.scripps.edu) facilitated the conversion of crocin and phospholipid structures into the appropriate pdbqt format for docking. Docking simulations were executed using AutoDock Vina 1.1.2, allowing crocin to remain flexible while treating the phospholipid as rigid. A grid map measuring 54 Å × 54 Å × 54 Å with a grid spacing of 0.375 Å was established to encompass the structures of crocin and phospholipid, using AutoDock Tools with the exhaustiveness number to 100. The docking type that resulted in the best binding energy scores was selected for further analysis. The interactions between crocin and the phospholipid and their visualization were thoroughly examined using Pymol^®^ software (The PyMOL Molecular Graphics System, Version 1.2r3pre).

#### 3.2.2. MD Simulation

The MD simulation was executed using the Gromacs package, version 2019. The 3D structures of crocin and phospholipid molecules were sourced from the DrugBank database (https://go.drugbank.com) and the Stockholm Phospholipids (Slipid) website (http://www.fos.su.se/~sasha/Slipids/Downloads.html), respectively. The general amber force field (GAFF) and the antechamber tool were employed during the simulation process to generate topology files for both crocin and phospholipid molecules. Subsequently, a suitable number of crocin and phospholipid molecules were placed in a cubic simulation box to achieve molar ratios of 1:1, 1:1.5, and 1:2. These prepared simulation boxes were then energy-minimized in the vacuum phase. Following this, the minimized systems were solvated with TIP3P water molecules, and energy minimization was performed again. Periodic boundary conditions (PBC) were applied in all three dimensions. After energy minimization, two equilibration steps were conducted: First in the NVT ensemble (constant temperature, constant volume) for 500 ps, followed by the NPT ensemble (constant temperature, constant pressure) for 500 ps, utilizing a V-rescale thermostat at 300 K and a Berendsen barostat at 1 bar pressure (17, 18). In the solvated systems, the heavy atoms of the crocin and phospholipid molecules were fixed and controlled with a force constant of 1000 kJ/mol/nm^2^ during the energy minimization steps. The Particle Mesh Ewald (PME) method was employed to calculate long-range electrostatic interactions (19), with the non-bonded cutoff distance set at 1.2 nm. The LINCS algorithm constrained all bonds involving hydrogen atoms (20). A production run was then carried out at a constant temperature and pressure for 100 ns, under conditions similar to those of the NPT equilibration step, saving atomic coordinates every 25 ps for subsequent analysis. The Gromacs modules analyzed the MD trajectories. Pymol software was utilized to visualize MD trajectories, generate molecular graphics images, and illustrate the interactions within the CPC.

### 3.3. Preparation of CPC

The CPC was synthesized using the solvent evaporation method (13, 21) with a molar ratio of 1:2 for crocin to EPC. Accurate amounts of crocin and EPC were fully dissolved in dichloromethane. The mixture was then refluxed in a 100 mL round-bottom flask at 40 - 45 °C for 3 hours. The resulting solution was evaporated under a vacuum at 40ºC using a rotary vacuum evaporator (Heidolph^®^, Germany) to remove the organic solvent. The obtained complex was collected, stored in an amber-colored glass bottle, and placed in the freezer for further analysis.

### 3.4. Physicochemical Characterization of CPC

#### 3.4.1. XRD

X-ray diffraction patterns for crocin, EPC, their PM, and CPC were obtained using an X-ray diffractometer (EQUINOX3000, Inel, France) equipped with Cu Kα radiation at a tube voltage of 40 kV and a current of 30 mA. The samples were scanned across an angular range from 5 to 80 °C 2θ at a scan rate of 0.03 °C 2θ/min.

#### 3.4.2. FTIR

Fourier-transform infrared (FTIR) spectra for crocin, EPC, PM, and CPC were recorded using a WQF-510 FTIR spectrophotometer (Rayleigh Analytical Instrument Corporation, China) within the wavenumber range of 4000 - 400 cm^-1^. The crocin powder was blended with potassium bromide (KBr) powder and then pressed into a disk. The other samples were dried under vacuum on a zinc selenide disk before undergoing FTIR analysis.

#### 3.4.3. NMR Spectroscopy

Proton nuclear magnetic resonance spectra for crocin, EPC, PM, and CPC were acquired on a Bruker Fourier 400 MHz spectrometer (Germany), using tetramethylsilane (TMS) as the reference standard. Each component was dissolved in deuterated dimethyl sulfoxide (DMSO-d6) for the analysis.

#### 3.4.4. FE-SEM

Field emission scanning electron microscopy (TESCAN, Czech Republic) at an acceleration voltage of 15 kV was employed to observe the morphological characteristics of crocin, EPC, their PM, and the CPC.

#### 3.4.5. Solubility Study

The solubility of crocin, PM, and CPC was assessed by adding excess amounts of each sample to specified quantities of water and n-octanol in sealed glass containers at room temperature. The samples were sonicated for 10 minutes, agitated on a magnetic stirrer for 24 hours, and then centrifuged for 10 minutes at 10000 rpm. The supernatant was diluted with water or methanol, and its concentration was measured using a UV-visible spectrophotometer (UV Mini 1240, Shimadzu, Japan) at 480 nm.

### 3.5. In Vitro Antioxidant Activity

The antioxidant activity of crocin and CPC was evaluated by their DPPH radical scavenging activity, with vitamin C (1 mg/mL) serving as a reference (22). Diverse concentrations of the samples (20 - 500 µg/mL) were mixed with DPPH solution and incubated for 30 minutes at room temperature in darkness. Absorbance was recorded at 492 nm using a microplate reader (Anthos 2020, Austria). The DPPH radical scavenging capability was calculated using the following equation:


$$DPPH\;scavenging\;activity\;\left(\%\right)=\;\frac{Abs.\;ofDPPH-(Abs.\;ofsample-Abs.\;ofblank)}{Abs.\;ofDPPH}\;\times 100$$

### 3.6. Statistical Analysis

The results were presented as mean ± standard deviation (SD). Statistical analysis was conducted using Student’s t-test or one-way analysis of variance (ANOVA), with a P-value < 0.05 indicating statistical significance.

## 4. Results

### 4.1. Molecular Docking

**Figure 2.**
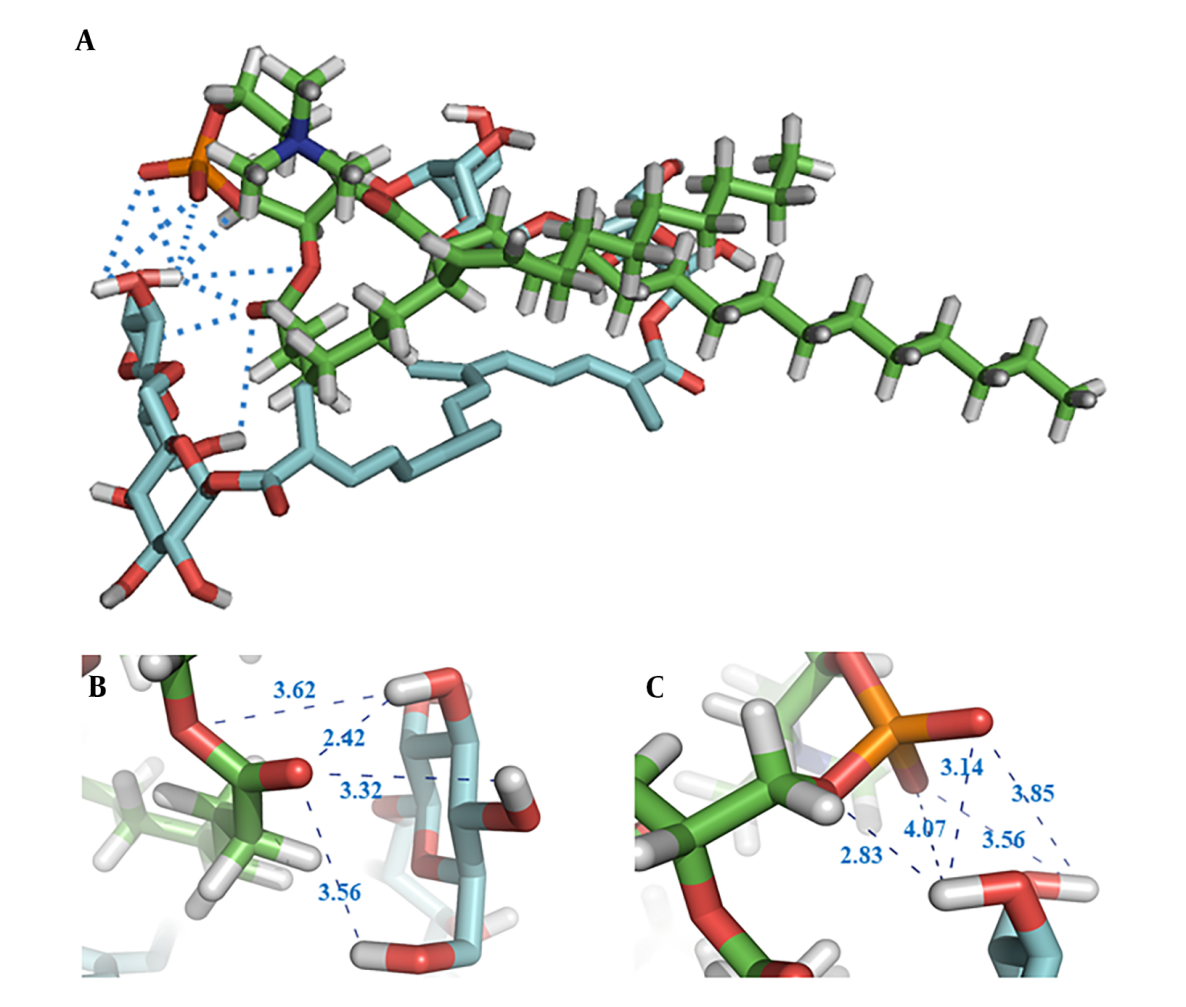
A, 3D conformation of crocin (with blue carbons) and phospholipid (with green carbons) post-docking, showcasing HB interactions between crocin's hydroxyl groups and the phospholipid's ester; B, and phosphate; C, groups.

The structure with the lowest energy, depicted in Figure 2A, shows the potential intermolecular hydrogen bond (HB) interactions between crocin and the polar head of the phospholipid, indicated by blue dotted lines. The HB distances observed in the docking study ranged from 2.42 Å to 4.07 Å. Additionally, the findings suggest that van der Waals forces and hydrophobic interactions are likely formed between the hydrophobic part of crocin and the two hydrophobic tails of the phospholipid. From Figure 2B and 2C, it is evident that the hydroxyl groups of crocin and the ester and phosphate groups of the phospholipid are involved in HB formation.

### 4.2. MD Simulation

**Figure 3.**
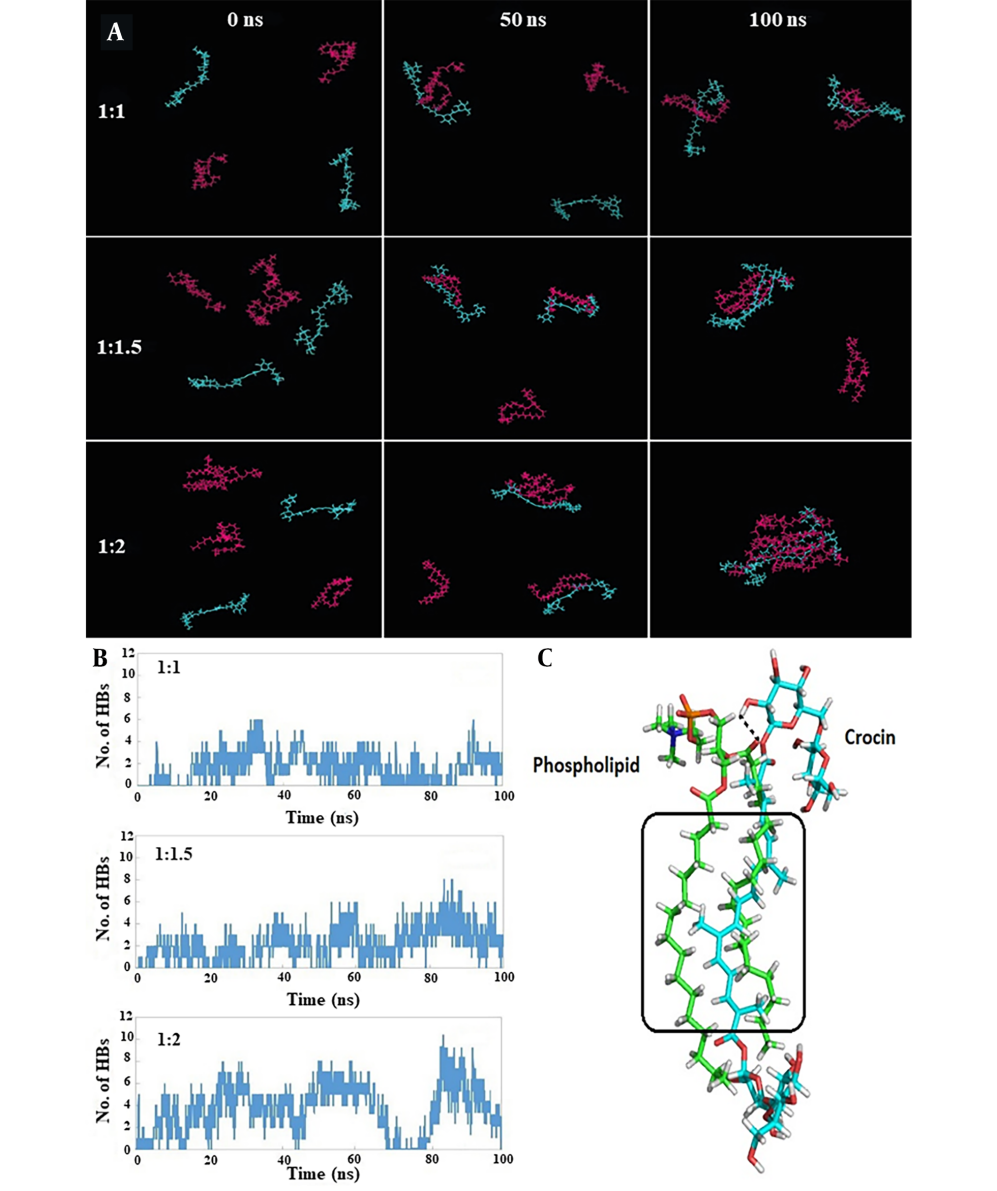
A, snapshots at 0, 50, and 100 ns demonstrating the spatial arrangement of crocin and phospholipid molecules next to each other during the simulation, with crocin (blue) and phospholipid (magenta) molecules shown in line representation; B, time evolution of hydrogen bond (HB) numbers between crocin and phospholipid molecules; C, detailed view of the orientation and interactions between a crocin molecule (blue carbons) and a phospholipid molecule (green carbons) in the 1:2 system, with HB and hydrophobic interactions marked by black dashed lines and black rectangles, respectively.

Table 1 presents the interaction energies calculated between crocin and phospholipid molecules for each simulation. The most stable and favorable interaction energy was associated with the 1: 2 crocin-to-phospholipid ratio, amounting to -429.9 kJ/mol. Figure 3A displays snapshots from the initial, middle, and final frames of the simulation, clearly illustrating the orientation of crocin and phospholipid molecules towards each other over the course of the simulation. In the 1: 1 system, as the simulation progresses, the crocin and phospholipid molecules draw closer so that by the simulation's end, each crocin molecule is positioned adjacent to a phospholipid molecule, similar to what is observed in the 1: 1.5 and 1: 2 systems. However, in the final frame of the simulation, the majority of crocin and phospholipid molecules are seen clustering together.

**Table 1.** Interaction Energy (Lennard-Jones, Coulombic, and Total) of the Simulated Systems (kJ/mol)

Simulated Systems	1: 1	1: 1.5	1: 2
**Lennard-Jones**	-265.3	-206.5	-232.4
**Coulombic**	-107.3	-129.7	-197.6
**Total**	-372.6	-336.2	-429.9

Figure 3B displays the evolution of HB numbers over time. It is evident that the 1: 2 and 1: 1 systems exhibited the highest and lowest numbers of HBs during the simulation, with average values of 3.2 and 1.6, respectively. The 1: 1.5 system, with an average value of 2.2, fell between these two.

The detailed orientation and interactions between crocin and phospholipid molecules in the 1:2 system are illustrated in Figure 3C. For clarity, one molecule of crocin and phospholipid was analyzed. An HB was identified between the hydroxyl group of crocin and the carbonyl group of the phospholipid's polar head. Additionally, hydrophobic interactions were noted, attributable to the hydrophobic regions present in both crocin and phospholipid molecules (highlighted by the black rectangle in Figure 3C). 

### 4.3. XRD

**Figure 4.**
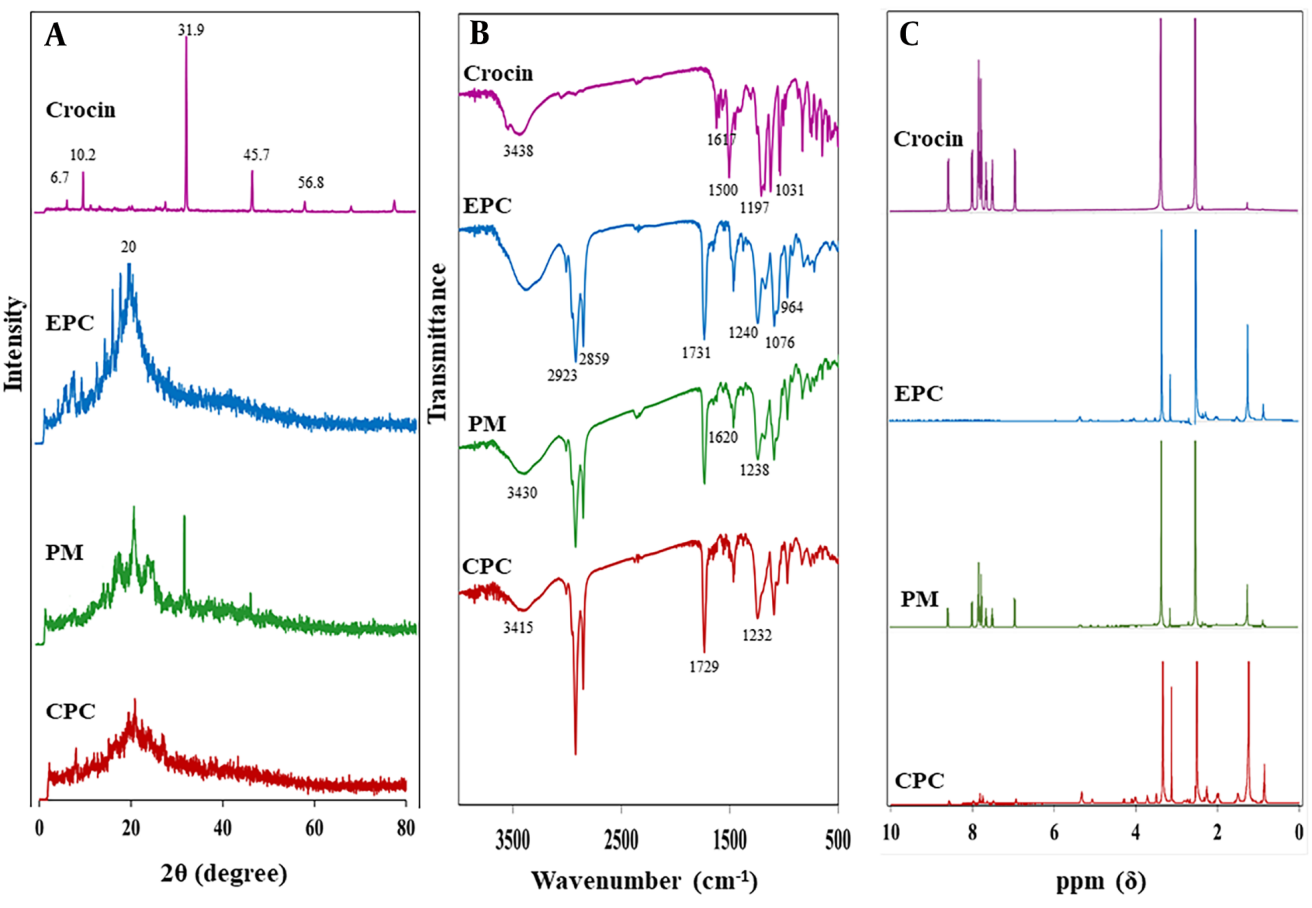
A, X-ray diffraction (XRD); B, Fourier-transform infrared spectroscopy (FTIR); C, ^1^H-NMR analyses of crocin, egg phosphatidylcholine (EPC), physical mixture (PM), and crocin-phospholipid complex (CPC).

Figure 4A presents the XRD patterns of crocin, EPC, their PM, and the CPC. Crocin's diffractogram displayed multiple sharp peaks at 2θ angles of 6.7°, 10.2°, 31.9°, 45.7°, and 56.8°, signifying its crystalline nature. The diffractogram of EPC showed a smooth, broad peak at around 20° 2θ, confirming its amorphous state. The PM exhibited characteristic sharp diffraction peaks of crocin with diminished intensity alongside a broad peak associated with EPC. In the CPC's XRD pattern, the prominent diffraction peaks of crocin were either absent or significantly reduced in intensity.

### 4.4. FTIR

The FTIR spectra for crocin, EPC, their PM, and the CPC are depicted in Figure 4B. The spectrum for crocin revealed characteristic peaks at 1617 cm^-1^ for C=O, 1500 cm^-1^ for C=C, 1197 cm^-1^ for C–O stretching vibration, and at 3438 cm^-1^ and 1031 cm^-1^ for O–H and C–O sugar groups respectively (23, 24). In EPC’s spectrum, the C–H stretching bands of the long fatty acid chains at 2923 and 2859 cm^-1^, the carbonyl stretching band at 1731 cm^-1^, the P=O stretching band at 1240 cm^-1^, the P–O–C stretching band at 1076 cm^-1^, and the N+(CH_3_)_3_ stretching at 964 cm^-1^ were observed (25). The PM’s spectrum displayed the primary frequencies of both crocin and EPC, with no or minor shifts in some frequencies. In the CPC's FTIR spectrum, notable changes were observed. The crocin-specific peaks at 1197 cm^-1^ (C–O stretching vibration) and 1031 cm^-1^ (C–O sugar groups) disappeared. Some peaks also showed chemical shifts. The transmission peaks for P=O and C=O stretching bands of EPC moved to lower wavenumbers at 1232 cm^-1^ and 1729 cm^-1^, respectively. Additionally, the peak for the O–H sugar group of crocin shifted to 3415 cm^-1^, showing a decrease in intensity and a broadening in shape.

### 4.5. NMR

The ^1^H-NMR spectra for crocin, EPC, PM, and CPC are presented in Figure 4C. The ^1^H-NMR spectrum for the PM resembled those of crocin and EPC. In the CPC’s ^1^H-NMR, the signals for EPC at 3.128 and 3.720 ppm shifted downfield to 3.129 and 3.722 ppm, respectively, attributed to the methyl proton of –N+(CH_3_)_3_ and the methylene proton of –CH2–N+. Moreover, the crocin signal at 3.356 ppm, representing the proton of the carbons attached to hydroxyl groups in the gentiobiose rings, shifted upfield to 3.343 ppm. Furthermore, the signals of crocin in the range of 7 to 9 ppm, indicative of the protons of the carbons forming the polyene (–HC=CH–), disappeared after complexation.

### 4.6. FE-SEM

**Figure 5.**
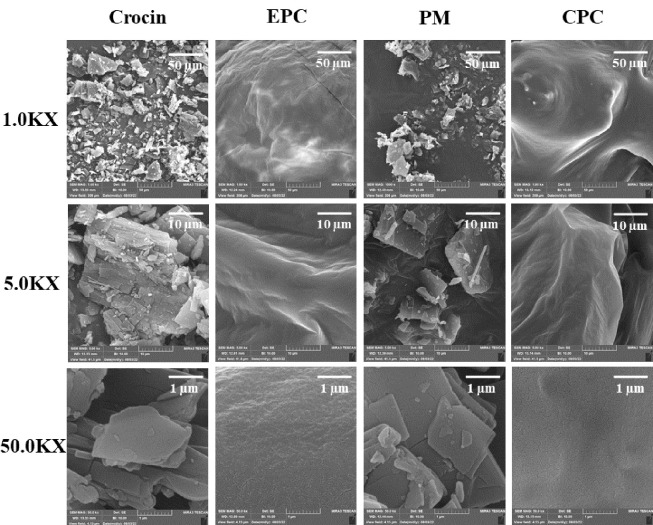
Field-emission scanning electron microscopy (FE-SEM) images of crocin, egg phosphatidylcholine (EPC), physical mixture (PM), and crocin-phospholipid complex (CPC) at various magnifications.

Field emission scanning electron microscopy was utilized to examine the surface morphology of crocin, EPC, their PM, and the CPC, as displayed in Figure 5. Pure crocin was characterized by rectangular-like crystals of smaller size, with regular shapes and smooth surfaces. EPC exhibited an irregular structure with a rough surface. The PM images showed both crocin and EPC retaining their original shapes. For the CPC, crocin's crystal structure was absent, and the particles assumed irregular shapes with smooth surfaces.

### 4.7. Solubility Study

**Figure 6.**
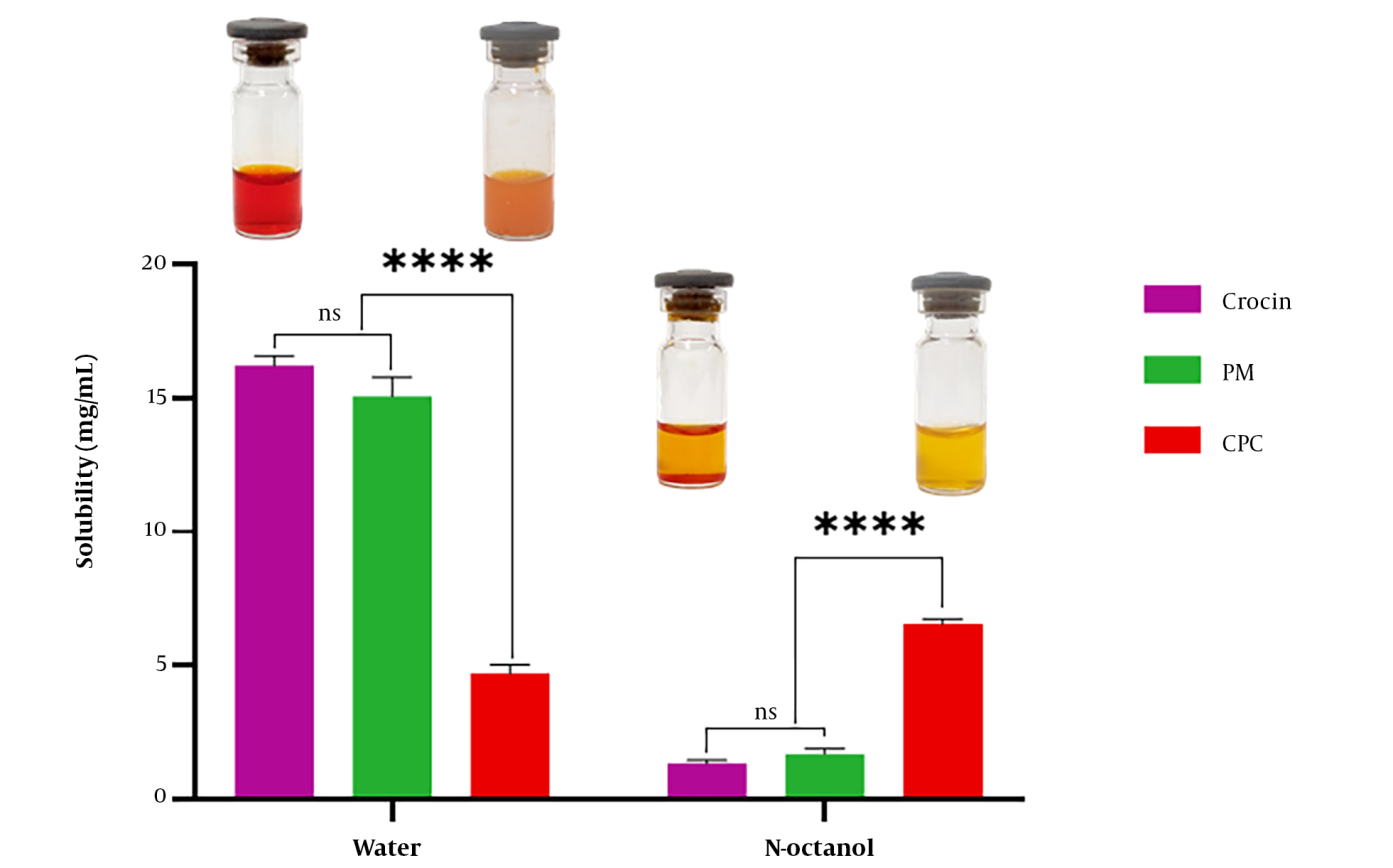
Solubility of crocin, physical mixture (PM), and crocin-phospholipid complex (CPC) in water and n-octanol (photographs of crocin and CPC in water and n-octanol are shown above their respective graphs)

Figure 6 illustrates the solubility data for crocin, PM, and CPC in water and n-octanol. The findings indicated that crocin's water solubility (16.21 ± 0.38 mg/mL) was approximately 3.5 times higher than that of CPC (4.70 ± 0.34 mg/mL), while the n-octanol solubility of CPC (6.56 ± 0.18 mg/mL) was around five times greater than that of crocin (1.36 ± 0.12 mg/mL). Additionally, the solubility of PM in water and n-octanol was similar to that of pure crocin without significant differences.

### 4.8. In Vitro Antioxidant Activity

**Figure 7.**
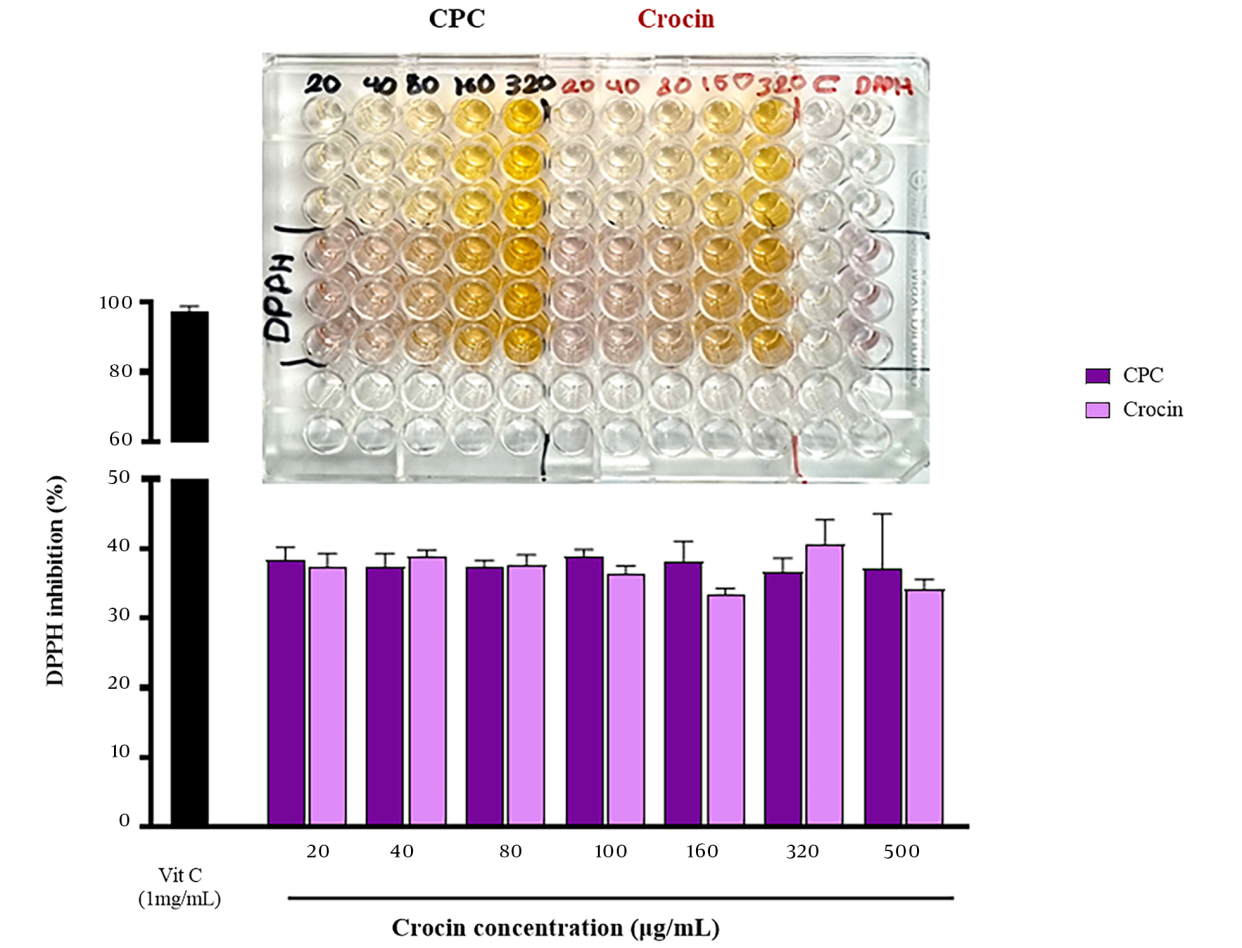
Antioxidant activity of crocin and crocin-phospholipid complex (CPC) (a photograph of the 96-well plate containing various concentrations of crocin and CPC incubated with diphenylpicrylhydrazyl (DPPH) is presented above the graph)

The antioxidant activities of crocin and CPC were 37.3% and 38.4% at 20 μg/mL and 34.15% and 37.1% at 500 μg/mL, respectively, as shown in Figure 7. There was no statistically significant difference in the DPPH radical scavenging activity between crocin and CPC across all tested doses and concentrations (n = 3, P > 0.01).

## 5. Discussion

The utilization of herbal drugs has seen an uptick in recent years, driven by their fewer side effects and greater patient compliance compared to synthetic medications. However, challenges such as low lipid solubility and high molecular weight have hindered their broader application. Phospholipid-based complex technology offers a viable solution to these limitations, enhancing the physicochemical properties of phytoconstituents and ensuring safety for pharmaceutical use due to the use of phospholipids as carriers (26, 27). This study focused on forming a complex between crocin, a water-soluble phytoconstituent, and phospholipids.

Molecular docking was initially employed to ascertain the affinity and potential interactions between crocin and EPC. This rapid, computer-based method provides a simulated 3D visual representation of binding sites and can predict the interactions between a ligand and a target (15, 28). The findings indicated potential for intermolecular HBs, van der Waals forces, and hydrophobic interactions between crocin and EPC during the complexation process. As depicted in Figure 2, the hydroxyl groups of crocin and the polar head of the phospholipid are within a distance of less than 4 Å, facilitating HB interactions with high likelihood. The molecular docking studies corroborated the ability of crocin to form a complex with EPC overall. These docking results were consistent with findings from FTIR and NMR analyses regarding the interaction types and sites. A similar approach was used in a study on the formation of a protopanaxadiol-phospholipid complex, employing Autodock Vina software for molecular docking analysis.

The conformation analysis results indicated that the hydrophobic part of protopanaxadiol was enveloped by the hydrophobic chains of the phospholipid. Additionally, an HB was formed between one of the hydroxyl groups in protopanaxadiol's structure and the phosphate group in the phospholipid's head (29). In another study, the interactions between rosuvastatin calcium and distearoylphosphatidylcholine (DSPC) were assessed through molecular docking. The calculated docking energy was -5.79 kcal/mol, demonstrating strong HB, van der Waals forces, and hydrophobic interactions between rosuvastatin and DSPC (30).

Given the significance of in silico methods for revealing molecular details of biological systems (31), MD simulation was utilized to explore the stability and potential interactions across different molar ratios of crocin and phospholipid to identify the optimal ratio. According to the findings (Table 1), the 1: 2 molar ratio exhibited the highest Coulombic interaction energy, attributed to a greater number of HBs formed between the crocin and phospholipid molecules. The HB analysis (Figure 3 and 3C) supported this result. Additionally, Figure 3A displays simulation snapshots showing that in all systems, the spatial arrangement of crocin and phospholipid molecules—particularly in the final simulation frame—facilitates maximum interaction between them. To our knowledge, this is the first study to employ MD simulation to determine the optimal molar ratio for creating a phospholipid complex. Previous studies have also reported the use of MD simulation for investigating the behavior, bonds, and stability of a metformin-phospholipid complex (32) and the configuration of a rosuvastatin-phospholipid complex in water (33).

Various tools are available to characterize drug-phospholipid complexes (12). In the present study, XRD, FTIR, NMR, FE-SEM, and solubility studies were conducted to evaluate and investigate the formation of CPC.

The XRD study reveals the crystalline nature of samples based on the existence or disappearance of large diffraction peaks or reduction in their intensity (34). As shown in Figure 4A, intense diffraction peaks of crocin support its crystallinity, while for the pure EPC, broad peaks indicate the presence of an amorphous structure. CPC exhibited no sharp peaks, indicating that crocin exists in the amorphous state within the complex due to its integration into the EPC and is no longer present as a crystalline material. These results are consistent with previous studies on the phospholipid complexes of erlotinib (21) and rifampicin (35). The sharp peaks related to erlotinib's crystalline structure were not observed in the diffractogram of its phospholipid complex (21), and the disappearance of rifampicin crystal peaks indicated its molecular dispersion in the amorphous phospholipid structure (35).

Infrared spectroscopy is a technique that predicts the formation of phospholipid-based complexes and their interactions based on functional groups and changes in characteristic peaks (12). The FTIR spectra of crocin and EPC were compared with earlier spectra (Figure 4B) (23-25). The PM spectrum was almost the summation of crocin and EPC peaks, while in the CPC spectrum, the disappearance or shifting of some peaks was observed. The most significant changes were shifts in the peaks of the crocin hydroxyl group (O-H) as well as phosphate (P=O) and carbonyl (C=O) groups of EPC, indicating the establishment of HBs between them. Similar changes have been reported in the FTIR spectra of chrysin (14) and tamoxifen (36) phospholipid-based complexes. Some changes were observed in the peaks related to the phenolic –OH and C=O groups of chrysin, along with the weakening of P=O and P-O-C peak signals of phospholipid, indicating their involvement in complex formation by HB interaction (14, 36).

^1^H-NMR spectroscopy, along with FTIR, is an important test to confirm complex formation (12). The ^1^H-NMR spectra of crocin and EPC were similar to those reported in previous studies (21, 37). As shown in Figure 4C, the spectrum of PM exhibited an additive effect of their individual components, while some changes were observed in the spectrum of CPC. Mild alterations in the chemical shift of characteristic protons of crocin and the polar head of EPC were observed, while the signal related to the non-polar segment of EPC remained constant. These changes indicate the interaction between crocin and the polar head of EPC to form CPC. These findings were consistent with the results reported for the erlotinib-phospholipid complex. Changes in the ^1^H-NMR spectrum of the erlotinib-phospholipid complex revealed the intermolecular interactions between erlotinib and phospholipid, indicating the formation of a stable complex (21).

The results of molecular docking, FTIR, and NMR studies are generally consistent with each other and demonstrate the successful formation of the complex. The slight differences in the results may be due to variations in the investigation medium; molecular docking was performed in a vacuum, while NMR was conducted in a DMSO-d6 solvent, and FTIR was performed in solid form.

In FE-SEM images of CPC (Figure 5), a significant change in morphology and shape was observed compared to pure crocin. Crystalline features of crocin disappeared, and the particles became amorphous. As mentioned previously in the study on the dihydromyricetin-phospholipid complex, this likely occurred due to the dispersal of crocin molecules in the phospholipidic matrix, leading to the formation of the complex (38).

Solubility study results (Figure 6) indicated that the complexation of crocin and phospholipid significantly increased the solubility of crocin in n-octanol. Crocin, having high aqueous solubility due to the presence of glycosyl in its structure (5), showed an enhancement in its lipophilic nature after complexation, which may improve its permeation through lipidic membranes. Another reason for the increase in the n-octanol solubility of crocin after complexation with phospholipid and the reduction of its crystallinity, leading to an amorphous state of the complex, is consistent with findings from studies on rutin-phospholipid complexes (39). These results align with conclusions drawn from studies on catechin-phospholipid complexes (40).

In recent years, many researchers have investigated the antioxidant effects of crocin, revealing protective effects against diabetes, cardiovascular dysfunction, cancer, and COVID-19 (41). In this study, the antioxidant activity of crocin and its complex was evaluated and compared at different concentrations. No significant difference was observed between the antioxidant ability of crocin and CPC (Figure 7), indicating that the antioxidant activity of crocin remained unchanged after complex formation. Similar findings were reported in studies on the antioxidant effects of quercetin and catechin and their complexes, where the formation of phospholipid complexes did not reduce the antioxidant activity of these herbal compounds (40, 42).

### 5.1. Conclusions

The phospholipid complex strategy can alter and enhance the physicochemical properties of phytochemicals, facilitating their application as herbal drugs. In this study, CPC was prepared and evaluated through in silico and physicochemical studies. Molecular docking predicted the binding affinity and formation of the complex, and MD simulation determined the most stable molar ratio of crocin and EPC for complex synthesis. Subsequently, CPC was prepared using the solvent evaporation method with the molar ratio obtained from MD simulation. The complex was characterized by XRD, FTIR, NMR, FE-SEM, and antioxidant studies. Solubility studies demonstrated that phospholipid complex technology could improve the lipid solubility of crocin. Thus, the phospholipid complex of crocin may enhance its permeation across lipidic biological membranes, warranting further validation through in vitro and in vivo studies.

## Data Availability

The dataset presented in the study is available on request from the corresponding author during submission or after publication.
